# Expression, purification, and characterisation of human soluble Epoxide Hydrolase (hsEH) and of its functional C-terminal domain

**DOI:** 10.1016/j.pep.2018.09.001

**Published:** 2019-01

**Authors:** Giancarlo Abis, Rebecca L. Charles, Philip Eaton, Maria R. Conte

**Affiliations:** a Randall Centre for Cell and Molecular Biophysics and British Heart Foundation Centre of Excellence, School of Basic and Medical Biosciences, King's College London, London, SE1 1UL, United Kingdom; b Cardiovascular Division and British Heart Foundation Centre of Excellence, The Rayne Institute, King´s College London, St Thomas' Hospital, London, SE1 7EH, United Kingdom

**Keywords:** Soluble epoxide hydrolase, *Escherichia coli*, Human embryonic kidney 293 free cells, Recombinant expression, C-terminal domain, EETs, epoxieicosatrienoic acids, AR9281, 1-(1-acetylpiperidin-4-yl)-3-adamantan-1-ylurea, AUDA, 12-[[(tricyclo[3.3.1.13,7]dec-1-ylamino)carbonyl]amino]-dodecanoic acid, GSK2256294, (1R,3S)-N-(4-cyano-2-(trifluoromethyl)benzyl)-3-((4-methyl-6-(methylamino)-1,3,5-triazin-2-yl)amino)cyclohexanecarboxamide, PHOME, 3-phenyl-cyano(6-methoxy-2-naphthalenyl) methyl ester-2-oxiraneacetic acid, 6M2N, 6-methoxy-2-naphtaldeyde, DTT, dithiothreiotol, TCEP, tris(2-carboxyethyl)phosphine, IMAC, immobilised metal affinity chromatography, SDS-PAGE, sodium dodecyl sulphate polyacrylamide electrophoresis, BTS, benzylthio-Sepharose, SEC, size exclusion chromatography

## Abstract

The human soluble Epoxide Hydrolase (hsEH) is an enzyme involved in the hydrolysis of endogenous anti-inflammatory and cardio-protective signalling mediators known as epoxyeicosatrienoic acids (EETs). EETs’ conversion into the corresponding diols by hsEH generates non-bioactive molecules, thereby the enzyme inhibition would be expected to enhance the EETs bioavailability, and their beneficial properties. Numerous inhibitors have been developed to target the enzyme, some of which are showing promising antihypertensive and anti-inflammatory properties *in vivo*. Thus far, the preparation of the recombinant enzyme for enzymatic and structural *in vitro* studies has been performed mainly using a baculovirus expression system. More recently, it was reported that the enzyme could be exogenously expressed and isolated from *E. coli*, although limited amounts of active protein were obtained. We herein describe two novel methods to yield pure recombinant enzyme. The first describes the expression and purification of the full-length enzyme from eukaryotic cells HEK293-F, whilst the second concerns the C-terminal domain of hsEH obtained from the cost-effective and rapid *E. coli* prokaryotic system. The two methods successfully generated satisfactory amounts of functional enzyme, with virtually identical enzymatic activity. Overall, the protocols described in this paper can be employed for the recombinant expression and purification of active hsEH, to be used in future biomedical investigations and for high-throughput screening of inhibitors for potential use in the treatment of cardiovascular disease.

## Highlights

• hsEH is a key regulator of cardiovascular homeostasis.
• A HEK293-F mammalian expression system for hsEH full-length (FL) was developed.
• An *E. coli* expression system for the hsEH C-terminal Domain (CTD) was established.
• Both proteins exhibited the same enzymatic specific activity *in vitro*.
• The CTD preparation provides benefits of easy operation, and high yield and purity.

## 1 Introduction

The human soluble Epoxide Hydrolase (hsEH – EC 3.3.2.10) is composed by two independently folded domains [[1], [2], [3]], an N-terminal domain (NTD) with phosphatase activity [3,4], and a C-terminal domain (CTD) which performs the hydrolysis of epoxy-fatty acids [3,[5], [6], [7]]. The CTD activity is involved in the homeostatic regulation of the cardiovascular tone, mainly through the hydrolysis of the epoxyeicosatrienoic acids (EETs), endothelial-derived hyperpolarising factors able to induce the relaxation of the vasculature in organs such as heart, kidneys, and many others [[8], [9], [10]]. The products of the EETs hsEH-mediated hydration, namely dihydroxyeicosatrienoic acids (DHETs), have a considerably reduced biological activity [10]. In the last 30 years, great efforts have been directed towards designing and synthesising effective inhibitors of hsEH CTD activity, with the ultimate aim to enhance the EETs’ bioavailability. This led to the discovery of several compounds able to reduce the enzyme hydrolytic activity *in vitro* with high potency and with promising *in vivo* efficacy in animal models of hypertension, atherosclerosis, diabetes, inflammation, pain, pulmonary disease and immunological disorders [11,12]. Of the thousands of compounds developed thus far, only few reached clinical trials. The two sEH inhibitor AR9281 [13] and AUDA [14] failed in a phase IIa study due to lack of efficacy [15,16]. Two phase I clinical trials with the GlaxoSmithKline compound GSK2256294 [17] have been recently completed. The results are still pending, even though no positive outcomes have been revealed yet [18,19]. As none of the thus far established antagonists exhibited absorption, distribution, and pharmacokinetics properties suitable for advanced clinical trials, the search for novel drug candidates is still an active field [12]. The rapid and cost-effective development of new compounds depends on the easy and manageable protocols of preparation of the hsEH. The great majority of the drug discovery studies on hsEH have been carried out producing the enzyme from *S. frugiperda* insect cells, via baculovirus recombinant expression [[20], [21], [22]]. However, this method is time-consuming and laborious. More recently, Nishi et al. [23] reported a protocol for the recombinant expression of the full-length human enzyme in *E. coli*. The purified enzyme showed virtually identical specific activity and inhibition properties of the recombinant enzyme prepared from insect cells. However, the yield of purified hsEH full-length (hsEH FL) obtained from prokaryotic cells was reported to be rather low (around 2 mg/L). Herein, we present a novel protocol of expression and purification of the recombinant hsEH FL, that yields milligrams of pure and active recombinant enzyme. This alternative method utilises the mammalian cells FreeStyle™ HEK293 (HEK293-F) [24]. Beyond the FL protein, Tanaka et al. [25] reported recently the first structure of the hsEH CTD, in complex with two novel inhibitors of the epoxy-hydrolytic activity of the enzyme, indicating that the CTD in isolation could be utilised to screen new libraries of hsEH antagonists. Although the CTD was obtained by recombinant expression in *E. coli*, very little detail on the protein expression and purification system was provided in the previous report. In this paper, we describe a thorough protocol of prokaryotic expression of the isolated CTD, along with an optimised purification method to yield milligram amounts per litre of pure protein for biological investigations and drug discovery studies.

In addition to protein cloning, expression, and purification of the hsEH FL and CTD, the enzymatic specific activity of the recombinant enzymes was also compared and will be herein described.

## 2 Materials and methods

### 2.1 Cloning of hsEH FL in mammalian expression vector

The cDNA of hsEH FL was kindly provided by Dr C Morisseau (UC Davis) and was cloned into the pcDNA3.1D/V5-His-TOPO^®^ vector (Invitrogen) using the pcDNA3.1 Directional TOPO^®^ Expression Kit (Invitrogen). The hsEH FL cDNA was PCR-amplified using primers designed to facilitate directional cloning (Table 1), as detailed in the kit instructions, generating a blunt-end PCR product that was mixed using a molar ratio of 1:1 of insert:TOPO^®^ vector. The mixture was then used to chemically transform One shot^®^ TOP10 E*. Coli* competent cells. Successful cloning was confirmed by sequencing (MWG Eurofins). The mammalian expression vector cloned with the hsEH FL cDNA was finally amplified in *E. coli* DH5α C2987 competent cells (NEB) and purified using the Pure Yield™ Plasmid Maxiprep system (Promega), out of a 0.5 L bacterial culture.

**Table 1:** Primers used in the cloning of the hsEH FL into pcDNA™3.1D/V5-His-TOPO^®^ vector. The primers were synthesised by Sigma.

Primer	Sequence
Forward	5′ **CACC**ATGACGCTGCGCGCGGCC 3′
	**Bold**: pcDNA™3.1D/V5-His TOPO^®^ annealing sequence to enable directional cloning
	Underlined: ATG initiation codon
	Normal: hsEH FL cDNA annealing sequence
Reverse	5′ CATCTTTGAGACCACCGGTGG 3′

### 2.2 Expression of hsEH FL in HEK293-F cells

A vial containing 1 × 10^7^ viable FreeStyle™ 293-F cells (HEK293-F – ThermoFisher Scientific) was transferred into a disposable Erlenmeyer flask (Sigma) previously filled with 30 mL of pre-warmed FreeStyle 293 Expression Serum-Free Medium™ (SFM – ThermoFisher Scientific). The cells were cultured for two days at 37 °C, 8% CO_2_, and 100 rpm, in a shacking incubator, up to a density of 1–3 × 10^6^ cells/mL and 90% viability. Cells were sub-cultured four times, every two days of incubation, in 30 mL of fresh SFM to 0.2–0.5 × 10^6^ cells/mL. During the fifth and last passage, the cells were seeded in 125 mL of fresh medium to 1 × 10^6^ cells/mL. After 48 h, the cells were prepared for transfection, being resuspended at a cell density of 2 × 10^6^ cells/mL in 250 mL of fresh pre-warmed SFM. The vector and the transfection agent polyethylenimine (Sigma) were added to the cells to a final concentration of respectively 3 μg/mL and 9 μg/mL. After 24 h, the culture was diluted 1:1 in pre-warmed SFM supplemented with 2.2 mM Valproic Acid (Sigma). Three days later the cells were collected by centrifugation at 1200 rpm for 20 min at room temperature and stored at −20 °C.

### 2.3 Cloning of hsEH CTD in bacterial expression vector

A modified pET3a plasmid, containing a hexahistidine tag (His-tag) and a TEV (tobacco etch virus) cleavage site (MHHHHHHSTENLYFQGSS) in the multiple cloning site, was provided by the Protein Production Facility of King's College London. The cDNA of the hsEH CTD (amino acids 230–555) was cloned in the plasmid using the IN-Fusion^®^ cloning ligation-independent system (Clontech Laboratories Inc.) [26]. The cDNA was PCR-amplified with the DNA polymerase KOD Hot Start Master Mix (EMD Millipore), using “chimeric” primers designed with the IN-fusion^®^ primer design web tool, characterised by a sequence that overlaps with the gene of interest, and a region complementary to the vector (Table 2). The template used was the mammalian expression vector containing the hsEH FL, described above. The plasmid pET3a was double digested for 3 h at 37 °C with BamHI/PstI (NEB), and the restriction enzymes were inactivated for 20 min at 80 °C. Plasmid and insert were mixed with a molar ratio of 1:3 respectively, and incubated with the In-Fusion HD Enzyme Premix for 30 min at 50 °C. The DNA was then used to transform DH5α C2987 E*. coli* competent cells. Successful cloning was confirmed by sequencing (Source Bioscience).

**Table 2:** Primers used in the IN-Fusion cloning of the hsEH CTD. The primers were synthesised by Sigma.

Primer	Sequence
Forward	5′ **ACCACAGCCA***GGATCC*AACCTCTTGCAATCCAAGTGA 3′
	**Bold**: pET3a annealing sequence
	*Italic*: BamHI restriction enzyme
	Normal: hsEH CTD cDNA annealing sequence
Reverse	5′ **GCAAGCTTGTCGAC***CTGCAG*TCATTACATCTTTGAGACCACCGGTG 3′
	**Bold:** pET3a annealing sequence
	*Italic:* PstI restriction enzyme
	Underlined: Stop Codons
	Normal: hsEH CTD cDNA annealing sequence

### 2.4 Expression of hsEH CTD in *E. Coli*

The pET3a containing hsEH CTD cDNA was transformed in chemically competent Ros2™(DE3) (EMD Millipore). A single colony of transformed cells was inoculated in 50 mL of ZYP5052 auto-induction medium [27] and cultured at 30 °C and 200 rpm overnight. The culture was diluted 1:100 in 3 × 1 L Erlenmeyer flasks filled up with fresh ZYP5052 medium and cultured at 37 °C and 200 rpm until the optical density of 0.5 was reached. The incubation temperature was then lowered to 18 °C and cells cultured for 24 h at 200 rpm. At the end of the incubation, the cells where centrifuged at 5000 × g for 30 min at 4 °C and the pellets stored at −20 °C.

### 2.5 Ni^2+^-IMAC chromatography

The pellets obtained from mammalian cells growths were resuspended in 5 mL/g of wet cells of lysis buffer (50 mM Tris-HCl pH 8, 300 mM NaCl, 10 mM imidazole, 5% glycerol, 2 mM PMSF, 2 mM MgCl_2_, 0.2 mM EDTA, 15 μg/mL benzamidine, a tablet of cOmplete™ protease inhibitor cocktail (Roche) per 50 mL of buffer), and the cells broken with three thaw-and-freeze cycles in liquid nitrogen. Pellets from bacterial cultures were resuspended in 2 mL/g of wet cells of lysis buffer (same as before, added with 0.01 g of lysozyme), to be sonicated on ice with a Vibra-Cell™ sonicator (Sonics) for 10 min (3 s pulse ON and 10 s pulse OFF). Both mammalian and bacterial lysates were then centrifuged at 38800 × g for 1 h at 4 °C, and the supernatants passed through a 0.45 μm filter (Millipore). The soluble fractions were loaded onto a 5 mL HisTrap™ FF Nickel Sepharose Column (GE Healthcare), previously equilibrated with binding buffer (50 mM Tris-HCl pH 8, 300 mM NaCl, 10 mM imidazole, 5% glycerol). The bound species were eluted in 1.5 mL fractions, applying a 0–60% gradient in 30 column volumes (CVs) of elution buffer (50 mM Tris-HCl pH 8, 300 mM NaCl, 300 mM imidazole, 5% glycerol). The fractions containing hsEH were pooled and dialysed at 4 °C overnight, in 20 mM MOPS pH 7.4, 75 mM NaCl, 1 mM EDTA, 2 mM DTT.

### 2.6 Benzylthio-Sepharose (BTS) chromatography

The Benzylthio-Sepharose (BTS) is an improved affinity chromatography resin for the purification of Epoxide Hydrolases. The resin was synthesised in-house using a protocol adapted from Winxtrom et al. [28]. 10 mL of CL-6B^®^ Sepharose resin (Sigma Aldrich) were poured onto a 15 mL polypropylene gravity column (Pierce), and washed with 10 CVs of water, 10 CVs of 50% MeOH, and 10 CVs of 0.1 M NaOH. The resin was then transferred in a 100 mL frosted neck round-bottom flask, containing 20 mL of 0.3 M NaOH, and 2 mL of 1,4-butanediol diglycidyl ether (activation solution), then cooled down for 30 min on ice while shaking. 60 mg of NaBH_4_ were added, and the epoxy-activation of the Sepharose was carried out at room temperature for 5 h on an orbital shaker in a fume hood (note that the flask was sealed with an aerated rubber stopper, to allow the release of the H_2_ generated by the reaction). The activated resin was transferred in a sintered-glass funnel and washed with 20 CVs of water pH 6, 20 CVs of 50% MeOH, 20 CVs of MeOH, 20 CVs of 50% MeOH, and 20 CVs of water pH 6. The resin was resuspended in 20 mL of activation solution, added with 50 mL of benzyl mercaptan, and left to swirl for 24 h at room temperature in a fume hood. The derived resin was transferred on a sintered glass funnel, and washed with 40 CVs of 50% MeOH, 40 CVs of MeOH, 40 CVs of 50% MeOH, 40 CVs of water, 40 CVs of 0.5 M NaCl, 40 CVs of water, 40 CVs of 0.001 M HCl to hydrolyse the unreacted epoxy-groups, 40 CVs of water, and 40 CVs of 50% EtOH. The resin was then split in aliquots of 2.5 mL in gravity columns and washed with 10 CVs of EtOH. The final BTS resin was stored at 4 °C in 0.1% of butylated hydroxyanisole.

Proteins subjected to dialysis after IMAC purification were loaded onto the BTS resin previously equilibrated with binding buffer (20 mM MOPS pH 7.4, 75 mM NaCl, 1 mM EDTA, 2 mM DTT, 5% glycerol). After washing the column with 2.5 CVs of binding buffer, hsEH proteins were eluted with 10 CVs of elution buffer (20 mM MOPS pH 7.4, 75 mM NaCl, 1 mM EDTA, 2 mM DTT, 5% glycerol, 1 mM 2-benzoyl-3-phenyloxirane). Purity and state of the protein were verified by SDS-PAGE, and the fractions containing protein were dialysed at 4 °C overnight in 137 mM NaCl, 2.7 mM KCl, 10 mM Na_2_HPO_4_, 3 mM KH_2_PO_4_, pH 7.4, 0.2 mM EDTA, 1 mM DTT.

### 2.7 Size-exclusion chromatography (SEC)

Protein samples were concentrated up to 5 mL, filtered with 0.2 μm filters (Millipore) and loaded onto a HiLoad 16/600 Superdex 75pg SEC (GE Healthcare), previously equilibrated with 137 mM NaCl, 2.7 mM KCl, 10 mM Na_2_HPO_4_, 3 mM KH_2_PO_4_, pH 7.4, 0.2 mM EDTA, 1 mM DTT, 5% glycerol. Fractions containing hsEH proteins were pooled together and dialysed overnight at 4 °C in 50 mM TRIS-HCl pH 7.4, 300 mM NaCl, 20% glycerol. After dialysis, samples were concentrated to 1 mg/mL (concentration adjusted according to the theoretical molecular weight and extinction coefficient, obtained using the ProtPARAM ExPASY tool [29]), flash frozen in small aliquots using liquid nitrogen, and stored at −80 °C.

### 2.8 Western blots

For immunoblotting, the samples were initially run on SDS-PAGE (using Laemmli protocol [30]) at 170 V for 60 min, to be then transferred onto a PVDF membrane (Sigma) for 1 h at 100 V (cooled with ice packs). The membranes were blocked with PBS and 10% milk, at room temperature in agitation for 1 h. After three washes of 10 min with 5 mL PBS-T (PBS, 0.1% Tween^®^-20), the membranes were exposed to the primary antibody in PBS-T added with 2% milk (mouse anti-6 × His-tag 1:5000 – Stratech CSB-MA000011M0m; rabbit anti-hsEH 1:1000 – Stratech CSB-PA007735LA01HU), shaken for 1 h at room temperature. After being washed three times for 10 min with 5 mL PBS-T, the membranes were exposed to HRP-conjugated secondary antibody in PBS-T added with 2% milk (goat-anti-mouse 1:5000 – Dako P0447; goat-anti-rabbit 1:2000 – Dako P0448). After 1 h in agitation at room temperature, the membranes were washed three times for 10 min, two times with 5 mL PBS-T, and a last time with PBS. The detection was carried out using the Enhanced Chemiluminescent Luminol (Promega).

### 2.9 Electrospray ionisation mass spectrometry (ESI-MS)

The intact protein MS experiments were performed using a maXis (Bruker) calibrated using the tuning mix ES-TOF (ESI) positive (Agilent). The proteins were dialysed overnight at 4 °C in 25 mM HEPES pH 7.4, 300 mM NaCl, 10% glycerol, 1 mM DTT, to be then concentrated to 5–10 mg/mL. The samples for ESI-MS were prepared diluting the proteins at a final concentration of 60 μg/mL in 0.1% formic acid, to be then loaded onto a Hamilton syringe and directly injected into the spectrometer using a motorised pump with a flow rate of 10–50 μL/min. The spectra were recorded for 3 min and a spectral rate of 1 Hz, with an end plate offset of −500 V, a capillary voltage of −4500 V, and a nebuliser pressure of 3.0 Bar. The detection of the spectra was performed setting the funnel and multipole RF at 400 Vpp, tuning the ISCID energy between 80 and 110 eV to optimise the signal-to-noise ratio, and selecting an ion energy of 3 eV.

### 2.10 Specific activity measurements

The enzyme specific activity was evaluated using a rapid continuous spectrofluorometric method developed by Wolf et al. [31]. The assay utilises the synthetic substrate 3-phenyl-cyano(6-methoxy-2-naphthalenyl) methyl ester-2-oxiraneacetic acid (PHOME – Cayman), which is hydrolysed by Epoxide Hydrolases, generating a detectable fluorescent end-product called 6-methoxy-2-naphtaldehyde (6M2N). The recombinant enzymes were thawed on ice and diluted with reaction buffer (25 mM TRIS-HCl pH 7.4) to 0.2, 0.4, 0.8, 1.6, 4, 8 μM concentrations, in previously cooled tubes. The aliquots were mixed with 10 μM TCEP for 15 min on ice and diluted 1:40 v/v in reaction buffer in a 96-well polystyrene microtiter plate (Thermo Scientific). A fresh 0.4 mM solution of PHOME was prepared in DMSO, to be then diluted 1:40 v/v in the 96-well plate. The measurements of the relative fluorescence units (RFUs) were carried out for 20 min using a POLARstar Omega (BMG Labtech), with the following setup: excitation wavelength 330 nm, emission wavelength 460 nm, detection every 45 s, gain 750, and 30 °C constant temperature. Measurements were performed in four replicates, and each experiment was repeated three times. For quantification purposes, a conversion curve was built reporting the 6M2N fluorescence as function of the fluorophore concentration. A series of 6M2N (Sigma) solutions with concentrations ranging between 0.01 and 10 mM were prepared in DMSO and diluted 1:25 v/v into reaction buffer. These solutions were then diluted 1:4 v/v into buffer in the 96-well plate and an endpoint fluorescence measurement was performed (same setting as before). The correlation between the fluorescence detected and the nmol of 6M2N was calculated by linear regression, and the slope used to convert the RFUs of the specific activity measurements into moles of product. Upon determination of the specific activity, the Turnover Frequency (TOF) was calculated. The hsEH FL and CTD TOFs were compared with one-tailed homoscedastic *t*-test and reported in the plots as average ± SEM. The statistical significance was set up at minimum *p* 0.05.

## 3 Results and discussion

### 3.1 Cloning of hsEH FL in TOPO pcDNA™3.1

Based on the cDNA sequence of the hsEH FL kindly provided by Dr C Morisseau and the instructions of the pcDNA3.1 Directional TOPO^®^ Expression Kit, a set of primers were designed (Table 1) to PCR-amplify the region corresponding to the hsEH FL cDNA. The amplification was confirmed by electrophoresis. The TOPO cloning reaction was set up mixing the blunt ended PCR product obtained and the pcDNA™3.1D/V5-His TOPO^®^ vector. After transformation of the One shot^®^ TOP10 E*. Coli* competent cells, confirmation of successful cloning was attained by sequencing of cDNA extracted from one of the colonies obtained (Supplementary material A).

### 3.2 Cloning of hsEH CTD in pET3a

Based on the cDNA sequence of the hsEH FL, a set of “chimeric” primers were designed (Table 2) to PCR-amplify the region corresponding to hsEH CTD cDNA (amino acids 230–555). The amplification of the PCR product, as well as the successful double digestion of the pET3a vector with BamHI/PstI were confirmed by electrophoresis. The IN-fusion reaction was set up and the mixture was transformed in homemade competent DH5α C2987 E*. coli* cells. The successful cloning was confirmed by sequencing of cDNA (Supplementary material B), extracted from one of the colonies obtained.

### 3.3 Optimisation of the expression conditions of hsEH CTD in *E. Coli*

Nishi et al. [23] previously reported a protocol of expression of hsEH FL in *E. coli* BL21(DE3) using the auto-induction media ZYP5052, given that induction with isopropyl-β-D-thiogalactopyranoside (IPTG) led to recombinant protein accumulation in inclusion bodies. We tested whether IPTG induction of Ros2™(DE3) cultured in Luria Broth or Terrific Broth produced insoluble hsEH CTD: in agreement with what was observed for the full-length recombinant enzyme [23], the CTD was also yielded exclusively in inclusion bodies at different induction temperatures, IPTG concentration and induction times (*data not shown*). However, using the autoinduction ZYP5052 media, the amount of hsEH CTD present in the soluble fraction increased noticeably. Transformed Ros2™(DE3) cells were cultured at 37 °C, up to three different levels of optical density (OD_600nm_) (0.5, 1, and 1.5), then the temperature was lowered to 18 °C and the cells cultured for either 16 or 24 h. Although most of the protein was still found in the insoluble fractions, detectable levels of soluble protein were observed when the cells were cultured at 37 °C up to an OD_600_ of 0.5, followed by 24 h incubation at 18 °C (Fig. 1). These optimised expression conditions were used for large-scale cultures. 2 L Erlenmeyer flasks were filled up with a volume of maximum 1 L of ZYP5052, to allow an adequate aeration of the medium during the long growth. Expression tests of the cultures were in agreement with the small-scale expression tests: most of the protein was insoluble, but detectable levels of soluble hsEH CTD were retained in solution.

**Fig. 1:**
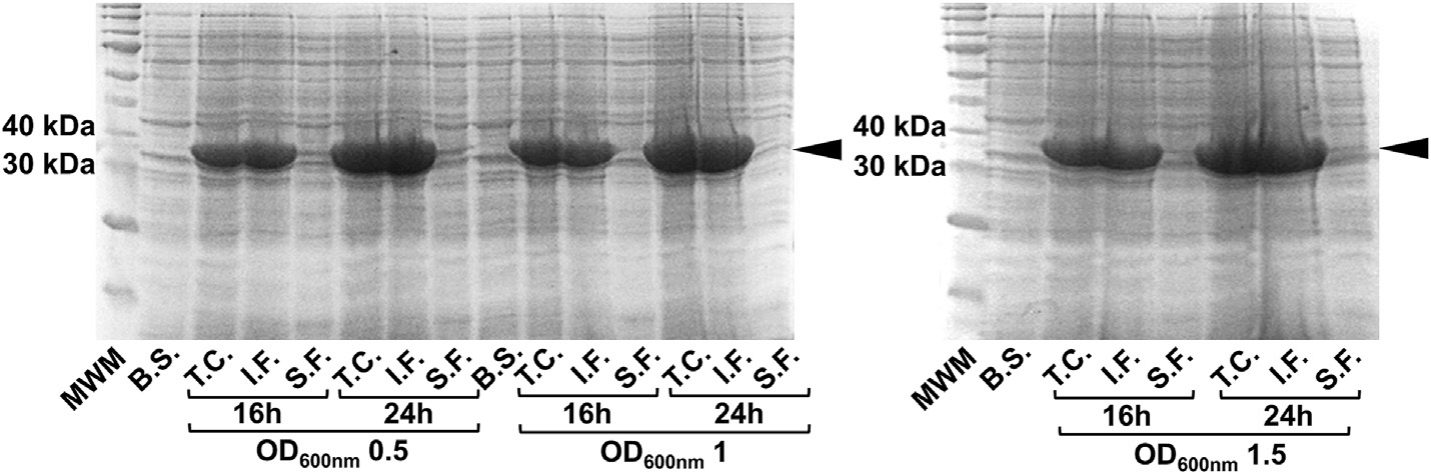
hsEH CTD expression tests in Ros2(DE3) *E. coli* cultured in ZYP5052 auto-induction media. hsEH CTD expressed well, with most of the protein being retained in the insoluble fractions. The best conditions for soluble proteins consisted in growing the cells at 37 °C up to an OD_600nm_ of 0.5, followed by 24 h at 18 °C (eighth lane from left). MWM: molecular weight marker; B.S.: before temperature switch; T.C.: total cells; I.F.: insoluble fraction; S.F.: soluble fraction; OD_600nm_: optical density at 600 nm.

### 3.4 Purification of hsEH FL

**Fig. 2:**
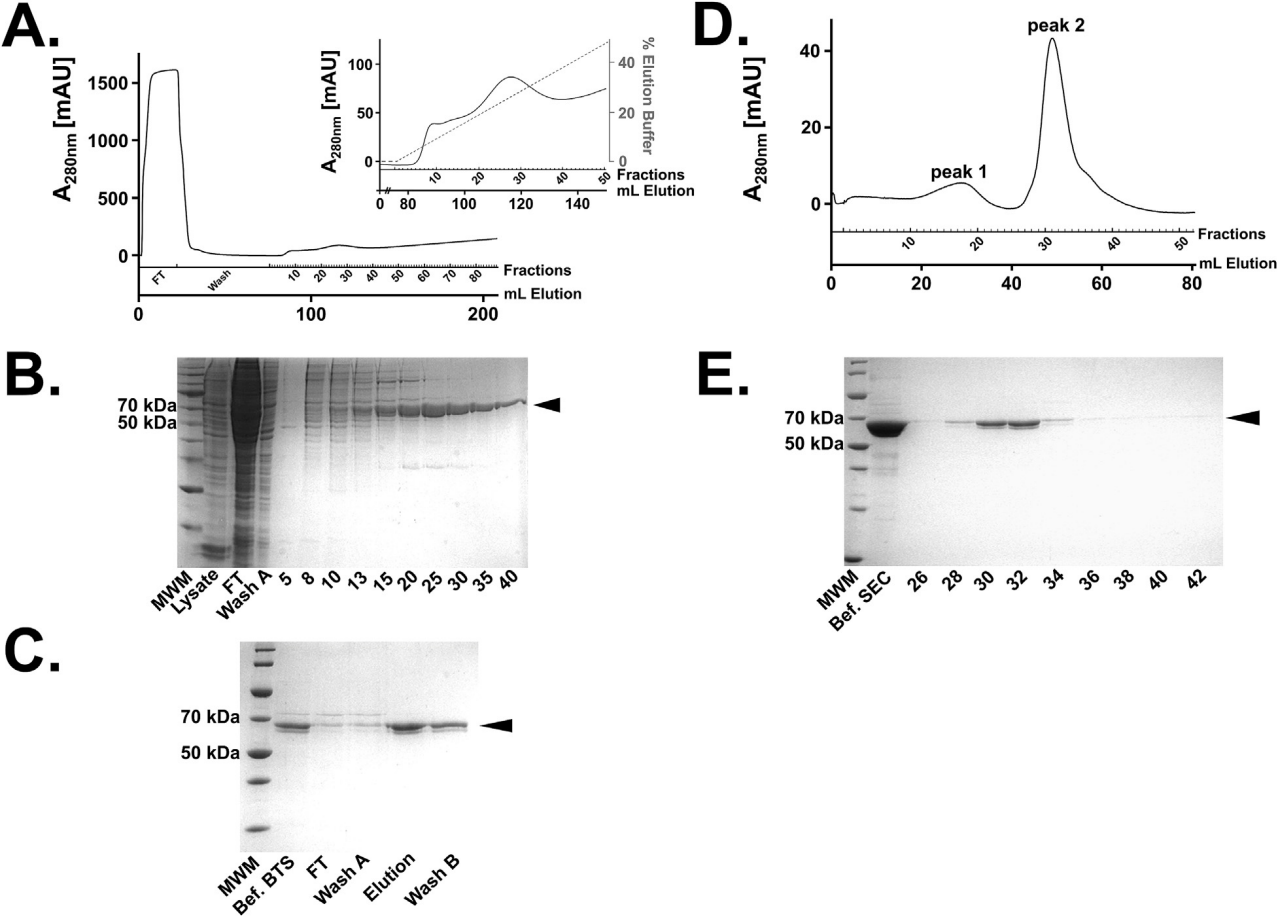
Purification of hsEH FL. **A.** IMAC elution profile. The elution produced two peaks, distinguishable between fractions 5 and 42. **B.** SDS-PAGE analysis of IMAC. The second elution peak contained hsEH FL. **C.** SDS-PAGE analysis following BTS purification. Following IMAC, the sample resulted almost pure (as shown in the “Before BTS” lane). The BTS affinity purification removed the minor residual contaminants. **D.** SEC elution profile. The elution produced two peaks, between fractions 12 and 45. **E.** SDS-PAGE analysis of SEC fractions. The hsEH FL was eluted in the second SEC peak. MWM: molecular weight marker; FT: flow-through; Wash A: wash with binding buffer; Wash B: wash with elution buffer.

Upon transfection of HEK293-F cells and expression of recombinant hsEH FL, pellets of ∼5 g of dry cells were obtained by centrifugation and stored at −20 °C. The cells were broken, and the lysate prepared for the purification. Taking advantage of the His-tag engineered at the C-terminus of the protein, the first stage of the hsEH FL purification was a Nickel IMAC. Fig. 2A shows the elution profile of the cell lysate through the HisTrap™ FF Nickel Sepharose Column. The SDS-PAGE analysis of the eluted fractions revealed the presence of hsEH FL in the peak eluted at approximately 22% of elution buffer, corresponding to about 70 mM imidazole (Fig. 2B). The fractions containing the protein of interest were dialysed overnight and loaded onto the BTS column, an affinity resin able to bind hsEH through the interaction of its CTD and the benzyl mercaptan moieties immobilised on Sepharose units [28]. SDS-PAGE analysis showed an improvement in the purity of hsEH FL (Fig. 2C) following this second purification step. As a final purification step, SEC was performed with the aim of removing any residual contaminant and preparing the protein for biochemical analysis. The SEC chromatogram, shown in Fig. 2D, indicated that the protein eluted in two peaks. The first peak showed an elution volume above the void volume of the column used, suggesting that part of the protein aggregated. The second peak exhibited an approximate elution volume of 50 mL, and the SDS PAGE analysis (Fig. 2E) revealed that it contained the hsEH FL. The fractions corresponding to the former peak were pooled together, yielding around 2 mg of protein from only 250 mL of transfected HEK293-F cells.

### 3.5 Purification of hsEH CTD

**Fig. 3:**
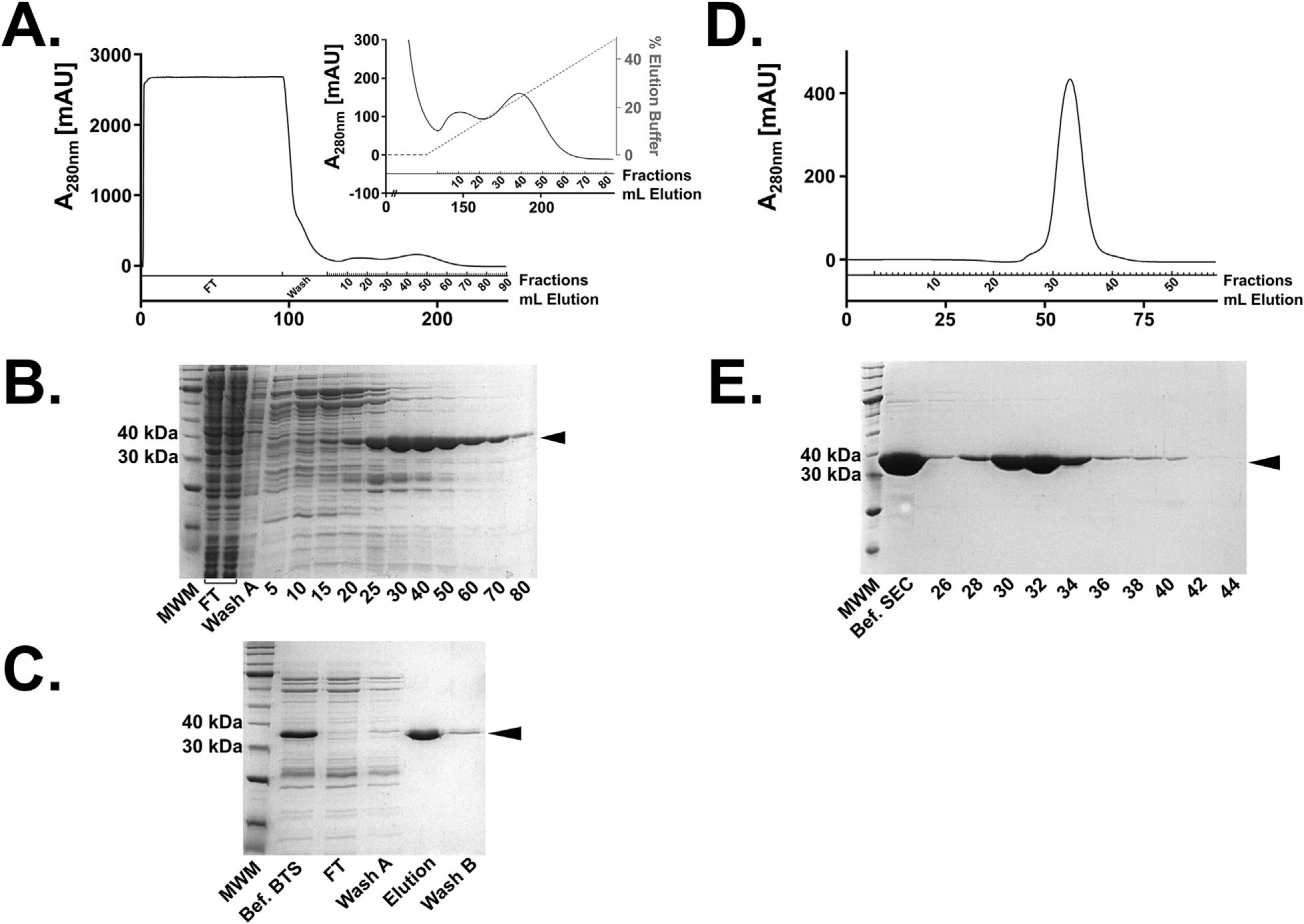
Purification of hsEH CTD. **A.** IMAC elution profile. The elution produced two peaks, distinguishable between fractions 5 and 75. **B.** SDS-PAGE analysis of IMAC fractions. The second elution peak contained hsEH CTD. **C.** SDS-PAGE analysis following BTS purification. The high affinity and specificity of BTS increased significantly the purity of the hsEH CTD sample. **D.** SEC elution profile. The hsEH CTD was eluted in a single peak between fractions 26 and 45. **E.** SDS-PAGE analysis of SEC fractions. The elution peak showed high purity levels and no signs of degradation.

After expression of hsEH CTD in Ros2™(DE3) cells, pellets of 10–15 g/L of cells were harvested. As per the full-length protein, the first step of purification was a Ni^2+^-IMAC, taking advantage of the His-tag engineered at the N-terminus of the construct. The elution profile showed two peaks (Fig. 3A), of which only the second contained hsEH CTD (Fig. 3B). The protein was then subjected to BTS affinity chromatography, obtaining a significant improvement of the purity (Fig. 3C). A final SEC chromatography step was performed revealing that hsEH CTD eluted in a single and sharp peak (Fig. 3E), indicative of a monomeric and monodisperse protein. The final sample showed high purity by SDS PAGE analysis (Fig. 3E). Approximately 20 mg of pure protein were yielded from 3 L of bacterial culture.

### 3.6 Characterisation of hsEH CTD and FL

**Fig. 4:**
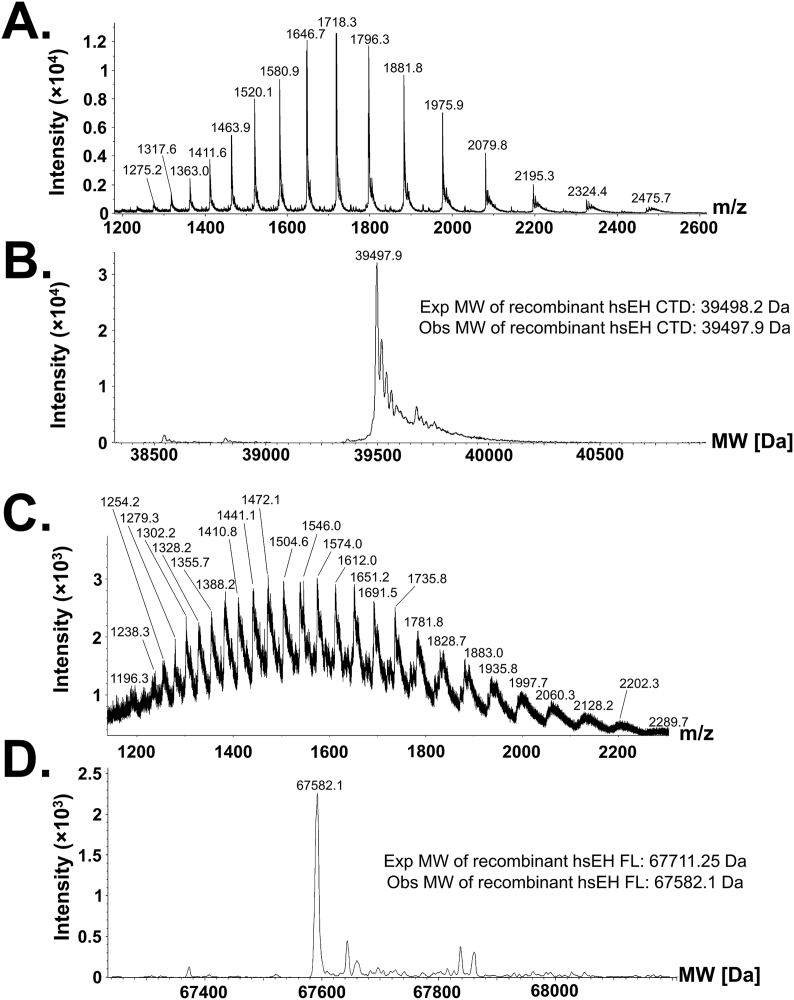
ESI-MS analyses of intact hsEH CTD and FL. **A.***m*/*z* detection of intact hsEH CTD. The *m*/*z* spectrum showed a Gaussian-type distribution of multiply charged ions ranging from *m*/*z* 1275.2 to 2475.7. **B**. Deconvolution of intact hsEH CTD *m*/*z* spectrum. The mass of the recombinant hsEH CTD was 39497.9 Da, virtually identical to the one computed with ProtParam. **C.***m*/*z* detection of intact hsEH FL. The *m*/*z* spectrum showed a Gaussian-type distribution of multiply charged ions ranging from *m*/*z* 1196.3 to 2289.7. **D.** Deconvolution of intact hsEH FL *m*/*z* spectrum. The deconvoluted mass of the recombinant hsEH FL was 67582.1 Da, corresponding to the molecular weight of the protein upon N-terminal methionine excision.

Electrospray ionisation mass spectrometry (ESI-MS) and Western blotting confirmed the identity of the purified proteins. The ESI-MS experiments were performed spraying hsEH CTD and FL in positive ionisation conditions, detecting the *m/z* intensity of the species in solution. The spectra showed a Gaussian-type distribution of peaks ranging from *m/z* 1275.2 to 2475.7 for the CTD, and 1196.3–2289.7 *m*/*z* for the FL (Fig. 4A and C). Through maximum entropy deconvolution, the plots shown in Fig. 4B and D were generated. In the case of hsEH CTD, the mass profile was dominated by a species with a molecular weight of 39497.9 Da, virtually identical to the expected mass of 39498.2 Da computed in ProtParam. The mass profile of hsEH FL was dominated by a species with a molecular weight of 67582.1 Da, corresponding to the expected mass of the recombinant protein upon N-terminal methionine excision [32] (67711.25 Da–131.19 Da = 67580.06 Da) (Fig. 4C). In addition to the main species, additional minor peaks at higher masses could be detected, attributed to counter ions bound to hsEH FL.

**Fig. 5:**
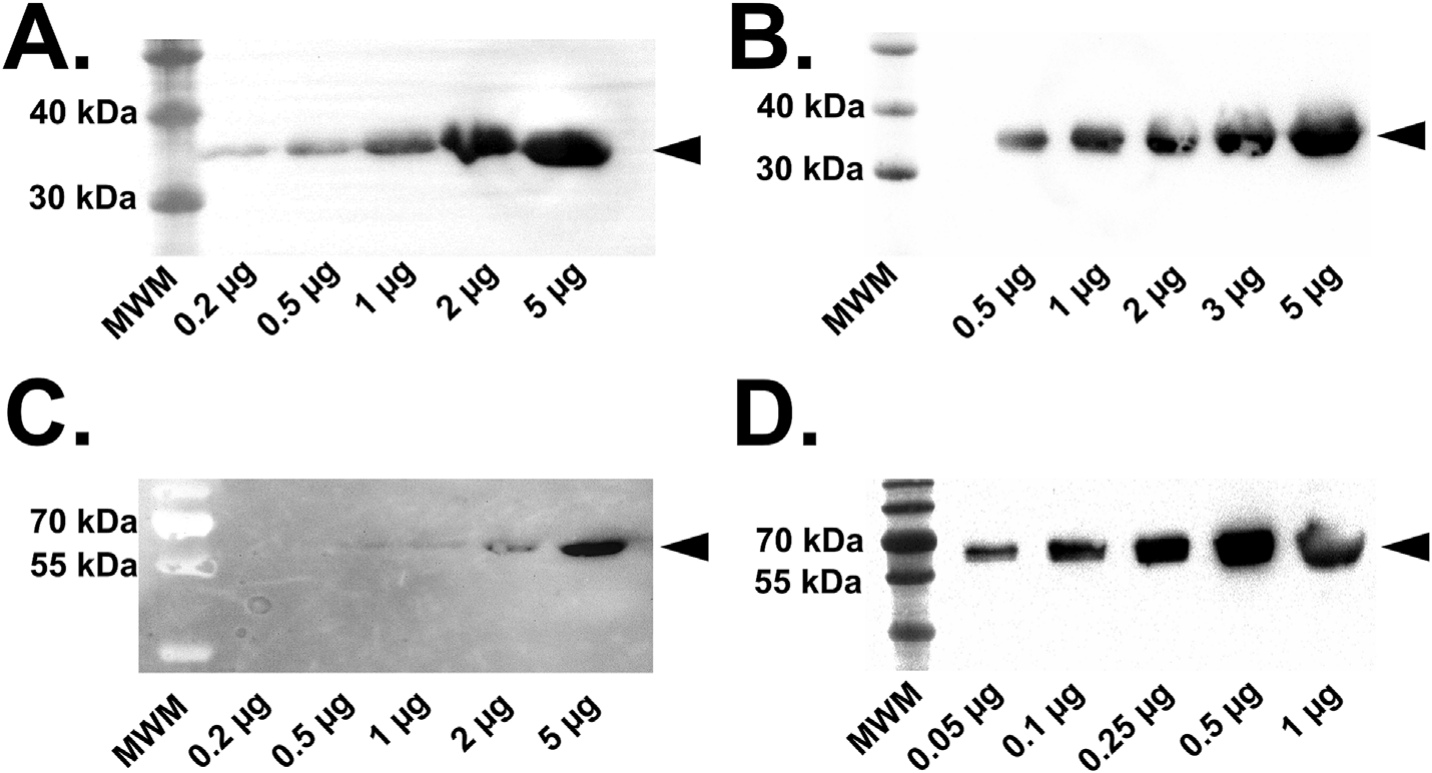
Western blotting analysis of hsEH CTD and FL. **A**. and **C.** Detection with anti-His-tag antibody for CTD and FL respectively. **B**. and **D.** Detection with anti-hsEH antibody for CTD and FL respectively. Signal detection of hsEH CTD and FL revealed only one band with both the antibodies employed.

Western blotting analyses were performed on purified hsEH CTD and FL, stained with two different primary antibodies: a mouse anti-His-tag monoclonal antibody (taking advantage of the His-tag engineered in the recombinant enzymes) (Fig. 5A and C) and a rabbit anti-human bifunctional Epoxide Hydrolase 2 (Fig. 5B and D). With both antibodies, it was detected only one band at the expected molecular weight for both hsEH CTD and FL, confirming the nature and the high purity of the protein samples.

Western Blot and ESI-MS experiments confirmed the identity of the recombinant hsEH CTD and FL expressed and purified from *E. coli* and HEK293-F cells respectively.

### 3.7 Specific activity measurements

**Fig. 6:**
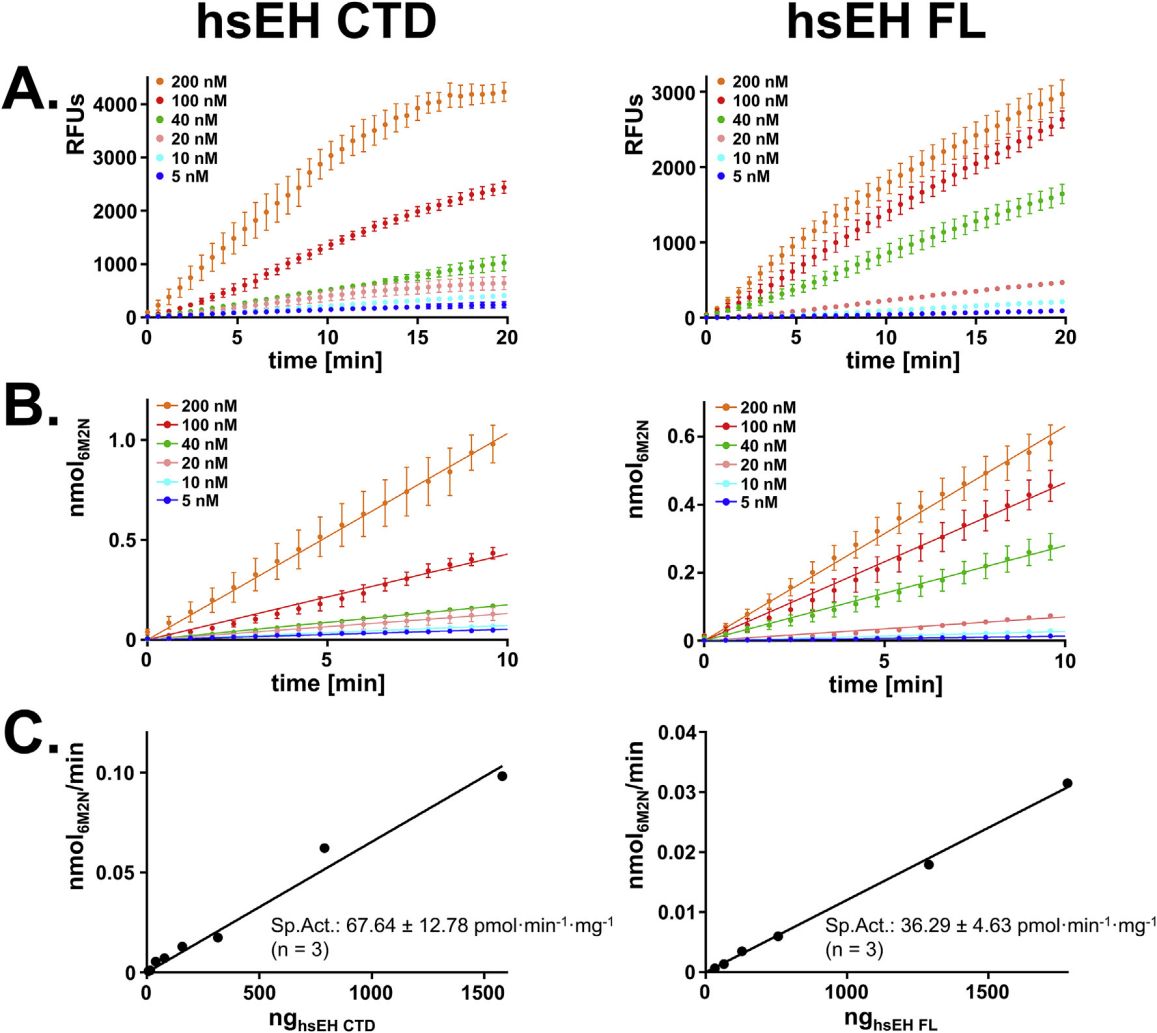
Enzymatic specific activity measurements of hsEH CTD and FL. **A.** Raw RFUs measurements. Increasing enzyme concentrations corresponded to growing fluorescence signal detection. **B.** Conversion to nmol of product vs. time of the RFUs linear region. The conversion was carried out using the slope of the conversion curve reported in Supplementary Material C. **C.** Specific activity determination. The parameter was calculated as the slope of the curve interpolated by the data points reported.

The specific activity of hsEH CTD and FL was determined *via* a spectrofluorometric assay, using the synthetic substrate PHOME [31]. Protein solutions with increasing concentrations of hsEH CTD and FL were prepared and diluted in the reaction buffer in adequate microplates. A saturating final concentration of the substrate PHOME was then added, and the fluorescence signal was monitored for 20 min. Fig. 6A reports the raw data obtained for hsEH CTD and FL, showing the expected fluorescence signal increase as function of protein concentration.

**Fig. 7:**
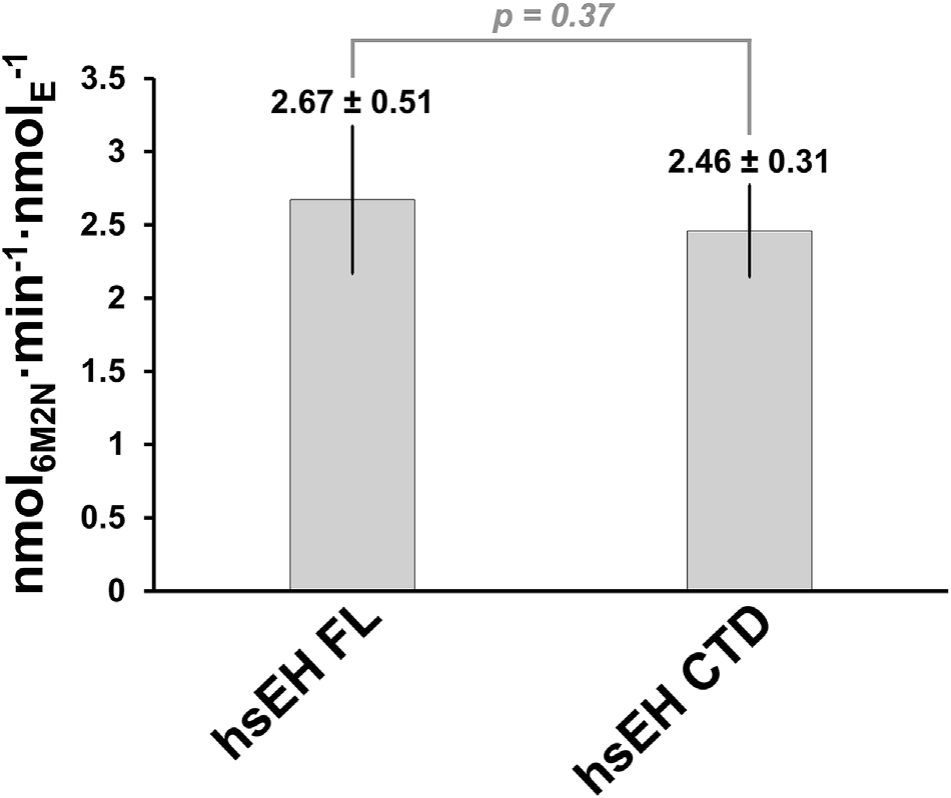
TOF comparison of hsEH CTD and FL obtained from the enzymatic specific activity. No significant difference of TOFs could be detected between the full-length enzyme and the CTD in isolation.

For accurate analysis, only the linear window of fluorescence increments between 0 and 10 min of detection was considered, corresponding to substrate-saturating conditions. The RFUs of the linear interval were transformed in nmol of 6M2N, using the conversion coefficient (Supplementary material C). The initial velocities of substrate conversion were determined as slopes of the straight lines obtained by linear regression (R^2^ ≥ 0.9) (Fig. 6B) and plotted versus the corresponding nmol of enzyme. The specific activity was determined by linear regression of the data points and further considered only when R^2^ ≥ 0.9 (Fig. 6C). The experiments were repeated at least three times, obtaining average specific activities of 67.64 ± 12.78 and 36.29 ± 4.63 pmol min^−1^·mg^−1^ for hsEH CTD and FL respectively. From the specific activity values obtained, the average TOF was calculated, to normalise by the number of available active sites, and compared with a one-tailed homoscedastic *t*-test (Fig. 7). The hsEH CTD and FL exhibited TOF of respectively 2.67 ± 0.51 and 2.46 ± 0.31 nmol min^−1^·nmol_enz_^−1^. The *t*-test did not reveal any significant difference of the average TOF of hsEH CTD and FL, as anticipated, as that the N-terminal Domain is not expected to be involved in the epoxy-groups hydrolysis [9].

## 4 Conclusion

hsEH is considered a key regulator of the cardiovascular homeostasis, a role exerted through the hydrolysis of endothelial signalling modulators termed epoxyeicosatrienoic acids (EETs) [8]. Accumulating evidence indicate that hsEH inhibition may be a potential strategy to increase the bioavailability of the EETs and enhance their beneficial cardioprotective activities. At the first stages of the hsEH inhibitors research, the enzyme used to be isolated from organs of different animal sources such as rats, mice, monkeys, and humans, through tissue homogenisation, ultracentrifugation, and enzymatic characterisation [33]. Such method was invaluable to understand localisation [34], function [35], and substrate specificity [36] of the mammalian sEH enzymes. Nonetheless, yields and purity were not sufficient to carry out structural or accurate biophysical investigations. It is only with the advent of sEH recombinant expression in *S. frugiperda* [37] that the first structure of murine sEH became available [1]. Since then, almost 100 structures have been deposited in the RCSB PDB, of both murine and human recombinant sEH, purified from insect cells. These structures showed many diverse compounds interacting with the enzyme, providing a precious tool for the characterisation and discovery of hsEH inhibitors. However, baculovirus protein expression is time-consuming and sometime technically challenging, welcoming the development of simpler and more efficient protocols of recombinant expression of the enzyme. Nishi et al. [23] reported a protocol of expression and purification of the full length hsEH from *E. coli* BL21(DE3) cells, obtaining around 2 mg/L of pure and active protein: nonetheless, in our hands, this method yielded even lower amounts of pure hsEH. We have therefore developed alternative expression and purification methods of both full-length protein and its functional C-terminal domain, which is necessary and sufficient for the EETs epoxy-hydrolytic activity of the enzyme. hsEH FL was obtained from mammalian HEK293-F cells, obtaining around 2 mg of protein from only 250 mL of culture. The optimised expression and purification of hsEH CTD using bacterial expression, the fastest and cheapest method of protein production available, yielded approximately 6 mg of pure protein per L of culture. Moreover, we herein demonstrated that the FL and CTD exhibited virtually identical turnover frequencies, suggesting that the CTD in isolation can be utilised in future studies of hsEH epoxy hydrolytic enzymatic activity and inhibition.

In conclusion, we have provided new tools to prepare active hsEH full-length or CTD, which will assist future drug discovery studies and investigations of its biological function and mechanisms.

## Author's contribution

All the authors contributed to the planning of the study. GA performed cloning and optimisation of bacterial expression of hsEH CTD, development and optimisation of purification of hsEH FL and CTD, characterisation of hsEH FL and CTD, and their specific activity measurements. RLC carried out cloning and optimisation of mammalian expression of the hsEH FL. GA, RLC, and MRC contributed to the writing of the manuscript. All the authors contributed to the results discussion. MRC and PE obtained funding for this work.

## Declaration of interest

Conflicts of interest: none.

## Figures and Tables

**figs1:**
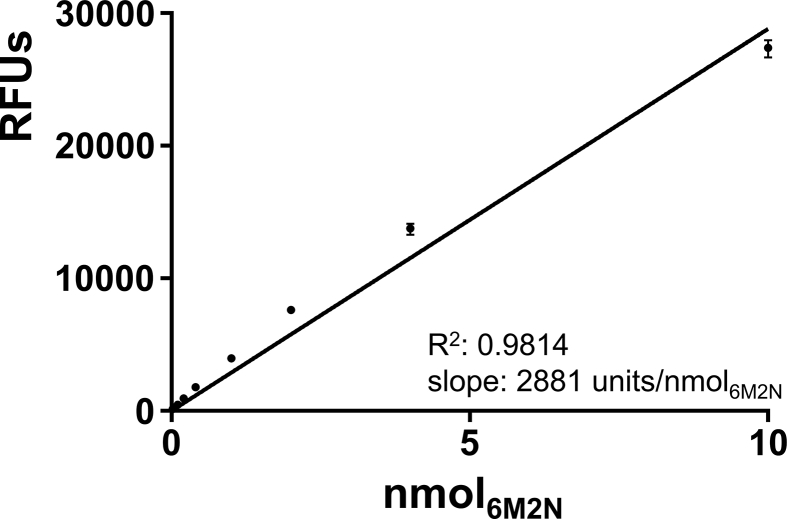


## Appendix A Supplementary data

Data Profile Data Profile
