# Supplementary material for: Expression, purification, and characterisation of human soluble Epoxide Hydrolase (hsEH) and of its functional C-terminal domain
Source: Protein Expr Purif. 2019 Jan;153:105–13. doi: 10.1016/j.pep.2018.09.001 (PMC6189638; doi:10.1016/j.pep.2018.09.001)
Supplement: Multimedia Component [file mmc2.docx]

## Supplementary material A

### Sequencing results of hsEH FL in TOPO pcDNA^™^3.1

1 atg acg ctg cgc gcg gcc gtc ttc gac ctt gac ggg gtg ctg gcg

**M T L R A A V F D L D G V L A**

46 ctg cca gcg gtg ttc ggc gtc ctc ggc cgc acg gag gag gcc ctg

**L P A V F G V L G R T E E A L**

91 gcg ctg ccc aga gga ctt ctg aat gat gct ttc cag aaa ggg gga

**A L P R G L L N D A F Q K G G**

136 cca gag ggt gcc act acc cgg ctt atg aaa gga gag atc aca ctt

**P E G A T T R L M K G E I T L**

181 tcc cag tgg ata cca ctc atg gaa gaa aac tgc agg aag tgc tcc

**S Q W I P L M E E N C R K C S**

226 gag acc gct aaa gtc tgc ctc ccc aag aat ttc tcc ata aaa gaa

**E T A K V C L P K N F S I K E**

271 atc ttt gac aag gcg att tca gcc aga aag atc aac cgc ccc atg

**I F D K A I S A R K I N R P M**

316 ctc cag gca gct ctc atg ctc agg aag aaa gga ttc act act gcc

**L Q A A L M L R K K G F T T A**

361 atc ctc acc aac acc tgg ctg gac gac cgt gct gag aga gat ggc

**I L T N T W L D D R A E R D G**

406 ctg gcc cag ctg atg tgt gag ctg aag atg cac ttt gac ttc ctg

**L A Q L M C E L K M H F D F L**

451 ata gag tcg tgt cag gtg gga atg gtc aaa cct gaa cct cag atc

**I E S C Q V G M V K P E P Q I**

496 tac aag ttt ctg ctg gac acc ctg aag gcc agc ccc agt gag gtc

**Y K F L L D T L K A S P S E V**

541 gtt ttt ttg gat gac atc ggg gct aat ctg aag cca gcc cgt gac

**V F L D D I G A N L K P A R D**

586 ttg gga atg gtc acc atc ctg gtc cag gac act gac acg gcc ctg

**L G M V T I L V Q D T D T A L**

631 aaa gaa ctg gag aaa gtg acc gga atc cag ctt ctc aat acc ccg

**K E L E K V T G I Q L L N T P**

676 gcc cct ctg ccg acc tct tgc aat cca agt gac atg agc cat ggg

**A P L P T S C N P S D M S H G**

721 tac gtg aca gta aag ccc agg gtc cgt ctg cat ttt gtg gag ctg

**Y V T V K P R V R L H F V E L**

766 ggc tcc ggc cct gct gtg tgc ctc tgc cat gga ttt ccc gag agt

**G S G P A V C L C H G F P E S**

811 tgg tat tct tgg agg tac cag atc cct gct ctg gcc cag gca ggt

**W Y S W R Y Q I P A L A Q A G**

856 tac cgg gtc cta gct atg gac atg aaa ggc tat gga gag tca tct

**Y R V L A M D M K G Y G E S S**

901 gct cct ccc gaa ata gaa gaa tat tgc atg gaa gtg tta tgt aag

**A P P E I E E Y C M E V L C K**

946 gag atg gta acc ttc ctg gat aaa ctg ggc ctc tct caa gca gtg

**E M V T F L D K L G L S Q A V**

991 ttc att ggc cat gac tgg ggt ggc atg ctg gtg tgg tac atg gct

**F I G H D W G G M L V W Y M A**

1036 ctc ttc tac ccc gag aga gtg agg gcg gtg gcc agt ttg aat act

**L F Y P E R V R A V A S L N T**

1081 ccc ttc ata cca gca aat ccc aac atg tcc cct ttg gag agt atc

**P F I P A N P N M S P L E S I**

1126 aaa gcc aac cca gta ttt gat tac cag ctc tac ttc caa gaa cca

**K A N P V F D Y Q L Y F Q E P**

1171 gga gtg gct gag gct gaa ctg gaa cag aac ctg agt cgg act ttc

**G V A E A E L E Q N L S R T F**

1216 aaa agc ctc ttc aga gca agc gat gag agt gtt tta tcc atg cat

**K S L F R A S D E S V L S M H**

1261 aaa gtc tgt gaa gcg gga gga ctt ttt gta aat agc cca gaa gag

**K V C E A G G L F V N S P E E**

1306 ccc agc ctc agc agg atg gtc act gag gag gaa atc cag ttc tat

**P S L S R M V T E E E I Q F Y**

1351 gtg cag cag ttc aag aag tct ggt ttc aga ggt cct cta aac tgg

**V Q Q F K K S G F R G P L N W**

1396 tac cga aac atg gaa agg aac tgg aag tgg gct tgc aaa agc ttg

**Y R N M E R N W K W A C K S L**

1441 gga cgg aag atc ctg att ccg gcc ctg atg gtc acg gcg gag aag

**G R K I L I P A L M V T A E K**

1496 gac ttc gtg ctc gtt cct cag atg tcc cag cac atg gag gac tgg

**D F V L V P Q M S Q H M E D W**

1541 att ccc cac ctg aaa agg gga cac att gag gac tgt ggg cac tgg

**I P H L K R G H I E D C G H W**

1586 aca cag atg gac aag cca acc gag gtg aat cag atc ctc att aag

**T Q M D K P T E V N Q I L I K**

1631 tgg ctg gat tct gat gcc cgg aac cca ccg gtg gtc tca aag atg

**W L D S D A R N P P V V S K M**

1676 aag ggt caa gac aat tct gca gat atc cag cac agt ggc ggc cgc

K G Q D N S A D I Q H S G G R

1721 tcg agt cta gag ggc ccg cgg ttc gaa ggt aag cct atc cct aac

S S L E G P R F E **G K P I P N**

1766 cct ctc ctc ggt ctc gat tct acg cgt acc ggt cat cat cac cat

**P L L G L D S T R** T G **H H H H**

1811 cat cat tga

**H H STOP**

**Bold**: protein sequence

**Grey:** His-tag
**Underlined:** V5-epitope

## Supplementary material B

### Sequencing results of hsEH CTD in pET3a

1 atg cat cac cat cac cat cac tcg acc gaa aac ctg tat ttt cag

M **H H H H H H** S T **E N L Y F Q**

46 ggc tcg agc acc tct tgc aat cca agt gac atg agc cat ggg tac

G S S **T S C N P S D M S H G Y**

91 gtg aca gta aag ccc agg gtc cgt ctg cat ttt gtg gag ctg ggc

**V T V K P R V R L H F V E L G**

136 tcc ggc cct gct gtg tgc ctc tgc cat gga ttt ccc gag agt tgg

**S G P A V C L C H G F P E S W**

181 tat tct tgg agg tac cag atc cct gct ctg gcc cag gca ggt tac

**Y S W R Y Q I P A L A Q A G Y**

226 cgg gtc cta gct atg gac atg aaa ggc tat gga gag tca tct gct

**R V L A M D M K G Y G E S S A**

271 cct ccc gaa ata gaa gaa tat tgc atg gaa gtg tta tgt aag gag

**P P E I E E Y C M E V L C K E**

316 atg gta acc ttc ctg gat aaa ctg ggc ctc tct caa gca gtg ttc

**M V T F L D K L G L S Q A V F**

361 att ggc cat gac tgg ggt ggc atg ctg gtg tgg tac atg gct ctc

**I G H D W G G M L V W Y M A L**

406 ttc tac ccc gag aga gtg agg gcg gtg gcc agt ttg aat act ccc

**F Y P E R V R A V A S L N T P**

451 ttc ata cca gca aat ccc aac atg tcc cct ttg gag agt atc aaa

**F I P A N P N M S P L E S I K**

496 gcc aac cca gta ttt gat tac cag ctc tac ttc caa gaa cca gga

**A N P V F D Y Q L Y F Q E P G**

541 gtg gct gag gct gaa ctg gaa cag aac ctg agt cgg act ttc aaa

**V A E A E L E Q N L S R T F K**

586 agc ctc ttc aga gca agc gat gag agt gtt tta tcc atg cat aaa

**S L F R A S D E S V L S M H K**

631 gtc tgt gaa gcg gga gga ctt ttt gta aat agc cca gaa gag ccc

**V C E A G G L F V N S P E E P**

676 agc ctc agc agg atg gtc act gag gag gaa atc cag ttc tat gtg

**S L S R M V T E E E I Q F Y V**

721 cag cag ttc aag aag tct ggt ttc aga ggt cct cta aac tgg tac

**Q Q F K K S G F R G P L N W Y**

766 cga aac atg gaa agg aac tgg aag tgg gct tgc aaa agc ttg gga

**R N M E R N W K W A C K S L G**

811 cgg aag atc ctg att ccg gcc ctg atg gtc acg gcg gag aag gac

**R K I L I P A L M V T A E K D**

856 ttc gtg ctc gtt cct cag atg tcc cag cac atg gag gac tgg att

**F V L V P Q M S Q H M E D W I**

901 ccc cac ctg aaa agg gga cac att gag gac tgt ggg cac tgg aca

**P H L K R G H I E D C G H W T**

946 cag atg gac aag cca acc gag gtg aat cag atc ctc att aag tgg

**Q M D K P T E V N Q I L I K W**

991 ctg gat tct gat gcc cgg aac cca ccg gtg gtc tca aag atg tga

**L D S D A R N P P V V S K M STOP**

**Grey:** His-tag
**Bold underlined:** TEV-cleavage site **Bold**: protein sequence

## Supplementary material C

### Conversion curve

The fluorescence detected by the spectrofluorometer in the specific activity measurements is determined in relative fluorescence units (RFUs). However, for quantification purposes the RFUs must be converted into moles of the fluorescent end-product of the reaction, namely 6-methoxy-2-naphthaldehyde (6M2N). For this purpose, solutions of the fluorescent product were prepared in DMSO at known concentrations and diluted with the reaction buffer in the microplates, to then detect their fluorescence at 460 nm. The correlation between the fluorescence detected and the nmol of fluorophore was calculated by linear regression (*Figure S1*). The slope of the curve represented the so-called conversion coefficient, used to transform the fluorescence units in nmol of 6M2N.

***Figure S1.*** RFUs conversion curve.
